# Characteristics of Infections in Hemodialysis Patients: Results from a Single-Center 29-Month Observational Cohort Study from Romania

**DOI:** 10.3390/microorganisms14010230

**Published:** 2026-01-19

**Authors:** Victoria Birlutiu, Rares-Mircea Birlutiu

**Affiliations:** 1 Faculty of Medicine, Lucian Blaga University of Sibiu, 550169 Sibiu, Romania; 2 County Clinical Emergency Hospital, 550245 Sibiu, Romania; 3 Department 14-Orthopedics, Anaesthesia Intensive Care Unit, Faculty of Medicine, “Carol Davila” University of Medicine and Pharmacy, 020021 Bucharest, Romania; 4 Foisor Clinical Hospital of Orthopedics, Traumatology, and Osteoarticular TB, 030167 Bucharest, Romania

**Keywords:** infections, hemodialysis patients, end-stage chronic kidney disease, outcome, clinical characteristics, pathogens, type of fistula

## Abstract

End-stage chronic kidney disease markedly increases susceptibility to infections due to compromised immune function and other physiological alterations. Bacteremia is responsible for higher mortality rates in hemodialysis patients compared to the general population. Our study aimed to investigate the incidence and clinical outcomes among patients with end-stage CKD and associated infections. The study retrospectively analyzed admitted patients between 1 January 2023 and 31 May 2025. Among 56 hospitalized patients with CKD and infection (30 hemodialysis [HD], 26 non-HD), baseline comorbidity profiles were broadly comparable. Microbiology was frequently positive (46/56, 82.1%), dominated by *Staphylococcus aureus* (25/98, 25.5%), *Klebsiella pneumoniae* (19.98, 19.4%), and *Escherichia coli* (15/98, 15.3%). Crude in-hospital mortality was higher in HD (46.7% vs. 15.4%; *p* = 0.012; RR 3.03). In multivariable logistic regression, HD remained independently associated with death (adjusted OR 38.22, 95% CI 1.55–940.53; *p* = 0.026), alongside hypotension (OR 17.55, 1.46–210.92; *p* = 0.024) and male sex (OR 4.41, 1.29–15.11; *p* = 0.018); model performance was strong (AUC 0.867). In this single-center cohort of infected patients with end-stage CKD, maintenance hemodialysis was independently associated with higher in-hospital mortality, even after adjustment for age, sex, comorbidity burden, hypotension, and length of stay; hypotension and male sex were additional risk factors. LOS and most presenting features did not differ meaningfully by dialysis status. Our findings also emphasize the urgent necessity for heightened surveillance of local antimicrobial resistance patterns and underscore the profound vulnerability of hemodialysis patients to severe infectious outcomes, which is exacerbated by immunosuppressive conditions and the limited efficacy of available therapeutic options against resistant pathogens.

## 1. Introduction

Patients with end-stage chronic kidney disease (CKD) require either hemodialysis (in most cases) or peritoneal dialysis (in the United States, for example, this occurs in 10% of cases) [1]. End-stage CKD is associated with a three-to-fourfold higher rate of infections compared with the general population. In 2017, this translated into 614 hospitalizations per 1000 individuals in the United States, compared with 214 hospitalizations per 1000 persons without acute renal insufficiency. Among infection-related admissions, sepsis, pneumonia, and urinary tract infections are the most frequently encountered. Understanding the etiological spectrum of infections in patients with mild to moderate CKD is as crucial as in those with end-stage disease, since effective antimicrobial therapy remains the cornerstone for achieving favorable patient outcomes [2].

Unfortunately, susceptibility to infections is high as a consequence of changes in both innate and acquired immunity, disturbances in phagocytosis, glycolysis, complement activation disorders [3], hyperparathyroidism, increased iron levels (which attenuate polymorphonuclear functions but possibly enhance bacterial virulence, promoting their multiplication) [4], etc., with infections being responsible for the second leading cause of death after cardiovascular diseases [4].

Several factors contribute to the increased risk of infection in patients with chronic kidney disease. These include arterial hypertension, diabetes mellitus [5,6,7], prolonged use of central venous catheters in hemodialysis patients, immunosuppressive therapy [8], malnutrition, hypoalbuminemia, anemia, and various forms of immunodeficiency. The latter may arise from functional alterations of polymorphonuclear cells, reduced counts of B lymphocytes, CD4+ T lymphocytes, monocytes, complement components, and dendritic cells [9,10], ultimately leading to impaired phagocytic activity [11]. Moreover, protection against certain vaccine-preventable diseases is often compromised, either due to low vaccine uptake or because of the rapid decline in protective immunity after vaccination [12].

The frequent exposure of patients to medical facilities is a significant risk factor for infections, as is patient-to-patient transmission of respiratory infections in particular, which are common during pandemics, such as influenza and SARS-CoV-2. This risk is associated with contact with surfaces, healthcare personnel, medical equipment, and *Staphylococcus aureus* colonization in patients and healthcare workers. Additionally, patients with central venous catheters and those undergoing dialysis are at risk of contamination owing to the presence of medical devices and possible contamination of dialysis water. Invasive medical procedures, such as injectable medication administration and skin barrier disruption, may also increase the risk of infection. It is also essential to consider bacterial virulence, as well as the tendency to increase antibiotic resistance among the pathogens involved. Tokars J. estimates that in the USA, the incidence rate of bacteremia in hemodialysis patients is 1.8% [13], while in France, bacteremia is considered to be the most frequent mode of infection progression, followed by respiratory, urinary, cutaneous, and soft tissue infections [14]. Bacteremia is responsible for mortality rates that are 100–300 times higher in hemodialysis patients compared to the general population, followed by pneumonia-related mortality, which has an even higher risk when considering the patient’s age, duration of hemodialysis, gender, and presence of comorbidities, with a particular emphasis on diabetes mellitus [15,16]. The highest risk is observed in patients undergoing dialysis via a central venous catheter [17], who may present catheter-related infections at a rate of 2.16% per month [18], which is ten times more frequent than in patients dialyzed via arteriovenous fistula [19].

In patients with end-stage renal CKD undergoing hemodialysis, the mortality rate is approximately 20% at one year and reaches 60% at five years [1,2]. The occurrence of an infectious complication further amplifies this risk, markedly increasing both hospitalization and mortality rates by nearly tenfold in the case of pneumonia and by up to one hundredfold in cases of sepsis [20].

An estimated glomerular filtration rate (eGFR) between 30 and 59 mL/min/1.73 m^2^ is associated with a 50% higher risk of hospitalization for infections compared with patients with an eGFR ≥60 mL/min/1.73 m^2^. Moreover, when eGFR declines to ≤30 mL/min/1.73 m^2^, the risk of infection-related hospitalization increases by two- to threefold [21,22,23].

Among the uremic toxins that contribute to the increased risk of infections through immune alterations, including leukocyte dysfunction, endothelial impairment, and aberrant macrophage activation, are indoxyl sulfate, p-cresyl sulfate, and trimethylamine-N-oxide (TMAO) [24,25,26,27,28,29,30,31,32]. In addition, oxidative stress and the accumulation of excessive reactive oxygen species (ROS) further exacerbate immune dysregulation [33,34].

Endothelial dysfunction is associated with increased levels of soluble markers, such as P-selectin, as well as glycocalyx degradation, which correlates with albuminuria and contributes to an elevated risk of infections [35,36,37].

Proinflammatory markers studied in patients with chronic kidney disease, including interleukin-6 (IL-6), tumor necrosis factor-alpha (TNF-α), and C-reactive protein (CRP), appear to be inversely correlated with the decline in glomerular filtration rate, thereby creating a proinflammatory state that contributes to an increased risk of infections [38,39,40].

Other factors that increase the risk of infections in patients with chronic kidney disease include disturbances in bone and mineral metabolism, particularly reduced levels of 25-hydroxyvitamin D and elevated fibroblast growth factor 23 (FGF23) [41,42].

From an etiological perspective, systemic infections in this category of patients are predominantly caused by *Staphylococcus aureus*, followed by coagulase-negative staphylococci, Gram-negative bacilli, and fungi.

**Figure 1:**
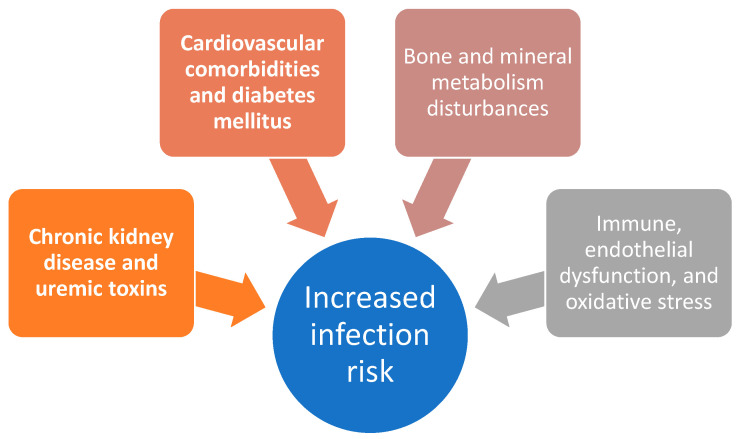
Schematic overview of selected mechanisms contributing to increased infection risk in patients with chronic kidney disease.

A graphical representation of the multifactorial pathways contributing to increased infection risk is highlighted in Figure 1.

Based on the previously mentioned data from the literature, this study investigated different factors associated with mortality in patients with chronic kidney disease. Accordingly, using data from a longitudinal observational cohort at a single institution, we conducted a retrospective study to characterize baseline and clinical features and to identify factors associated with adverse status at hospital discharge.

## 2. Materials and Methods

We performed a single-center longitudinal observational cohort study on patients with chronic kidney disease and associated infection that did or did not require hemodialysis who were hospitalized in Sibiu County Clinical Emergency Hospital, Romania. In these analyses, we retrospectively included all consecutive adults (≥18 years) admitted to the Infectious Diseases Clinic of the Sibiu County Clinical Emergency Hospital, Romania, between 1 January 2023 and 31 May 2025, with confirmed chronic kidney disease (including end-stage CKD) and who were hospitalized with a concurrent infectious episode. Extracted medical record data included demographic characteristics, risk factors and comorbidities, laboratory findings, administered treatments, in-hospital complications, and discharge outcomes. Data extraction and consistency checks were performed by both authors. Patients with acute kidney failure were excluded.

The main objective of our analysis was to evaluate the risk factors associated with an unfavorable outcome. The secondary objective of our analysis was to evaluate enrolled patients’ clinical and baseline characteristics.

Microbiological investigations were performed using standardized, protocol-driven procedures to ensure broad pathogen detection. The laboratory operated continuously (24/7), including weekends, enabling the timely processing of urgent specimens. Specimen type and testing were guided by the patient’s clinical syndrome. For all enrolled patients, at least two sets of blood cultures were obtained on the day of admission from separate venipuncture sites, 12 h apart, and were repeated during hospitalization when clinically indicated. Blood cultures were processed using an automated system (BACT/ALERT^®^ 3D with BACT/ALERT^®^ culture media; bioMérieux, Marcy-l’Étoile, France). When lower respiratory tract infection was suspected, sputum and/or tracheal aspirates were collected and cultured. As part of the routine quality assurance, sputum adequacy was assessed microscopically on a stained smear at low-power magnification to estimate oropharyngeal contamination. Samples were considered representative of lower respiratory tract infection when they contained few squamous epithelial cells and >25 leukocytes per low-power field, consistent with active inflammation. Respiratory specimens were collected in the morning under nursing supervision; patients were instructed to avoid antiseptic mouthwash and food intake prior to sampling. All specimens were incubated under aerobic, anaerobic, and capnophilic (CO_2_-enriched) conditions at 35–37 °C, using Schaedler anaerobe broth and additional selective/differential media as required, including fungal-specific media when indicated. Bacterial isolates were identified primarily using the VITEK 2 Compact system (bioMérieux, Marcy-l’Étoile, France). Where needed, additional methods were employed, including multiplex PCR with biotinylated primers and reverse-hybridization on membranes containing pathogen-specific probes. The laboratory also had access to MALDI-TOF MS (VITEK^®^ MS, bioMérieux, Marcy-l’Étoile, France) and 16S rRNA gene sequencing (Illumina MiSeq^®^ platform, Illumina, San Diego, CA, USA), which were applied in selected cases. Antimicrobial susceptibility testing was performed using fully or semi-automated platforms. Minimum inhibitory concentrations (MICs) were interpreted according to EUCAST breakpoints, and, in a limited number of historical cases, CLSI MIC criteria was used, which were valid at the time of testing.

All analyses were conducted in IBM SPSS Statistics (v29). Continuous variables were presented as median or mean (standard deviation) as appropriate, and categorical variables as counts and percentages. Normality was assessed using the Shapiro–Wilk and Kolmogorov–Smirnov tests. Between-group comparisons for non-normally distributed continuous variables were performed using the Mann–Whitney U test. Categorical variables were compared using the χ^2^ test or Fisher’s exact test, as appropriate; Cramér’s V was used to quantify the strength of association. A two-sided *p*-value < 0.05 was considered statistically significant.

A classification and regression tree (CART) model was constructed to identify factors associated with in-hospital death based on the results of the multivariable regression analyses.

All participants provided written informed consent prior to inclusion. The study was performed in accordance with the Declaration of Helsinki and received approval from the Institutional Ethics Committee (approval no. 11494; 9 May 2024); the Ethics Committee approved the use of anonymized data for publication.

## 3. Results

Of the hospitalized patients in the Infectious Diseases Clinic of the Sibiu County Clinical Emergency Hospital, Romania, between 1 January 2023 and 31 May 2025, 56 were diagnosed with chronic kidney disease and infections, and for all included cases in our analysis a complete electronic medical record was available. We aimed to retrospectively evaluate 30 patients with end-stage chronic kidney disease who required hemodialysis (HD group) versus 26 patients with end-stage chronic kidney disease who did not require hemodialysis (non-HD group).

In univariable χ^2^ screening of all the comorbidities, only hypotension remained significantly associated with hemodialysis status (*p* = 0.046), indicating a modest under-representation of this condition among dialyzed patients (7% vs. 31% in the non-HD group). Across the full cohort (*n* = 56) the total number of in-hospital days ranged from 2 to 68 (mean ± SD = 14.9 ± 14.1; median = 10). Advanced ventilatory support was uncommon and showed no statistical significant relationship with hemodialysis (HD) status. The use of an oxygen mask was documented in 14 patients (25.0%), which occurred slightly more often in the non-HD group (6/26 vs. 8/30; 30.8% vs. 20.0%; *p* = 0.35).

An overview of the enrolled patients is shown in the following table (Table 1).

In the contingency analysis that crossed hemodialysis (HD) status with the status at the time of discharge, hemodialysis was strongly associated with in-hospital mortality. Among non-HD patients, 22 recovered (84.6%) and 4 died (15.4%), whereas among the dialyzed patients, only 16 recovered (53.3%) and 14 died (46.7%). Pearson’s χ^2^ test yielded χ^2^(1) = 6.25, *p* = 0.012, and the continuity-corrected value remained significant (χ^2^ = 4.90, *p* = 0.027). These findings indicate that, in univariable terms, maintenance hemodialysis is associated with a markedly higher in-hospital death rate, warranting further multivariable adjustment to determine whether the effect is independent of age, comorbidity burden, and severity of illness at admission.

A detailed analysis of the laboratory tests performed is presented in Table 2. Sixteen laboratory indices were contrasted between non-HD patients and HD patients using the Mann–Whitney U test (values reported as mean).

In terms of clinical manifestations, our dataset contained information on various clinical manifestations at the time of admission to the department, such as fever, chills, cough, dyspnea, cardiac manifestations, urinary tract manifestation, digestive manifestations, skin lesions, and neurological manifestations. A detailed overview of the performed crosstabulations is presented in the following table (Table 3).

Overall, symptom prevalence was broadly comparable between groups, but several patterns emerged. Cough was less frequent in HD patients (11/30 vs. 17/26; 36.7% vs. 65.4%), *p* = 0.032, suggesting a lower respiratory symptom burden in HD. Similarly, urinary manifestations were markedly less common among HD patients (3/30 vs. 9/26; 10.0% vs. 34.6%; *p* = 0.025). In contrast, neurological symptoms were more frequently observed in HD patients (15/30 vs. 8/26; 50.0% vs. 30.8%), though this did not reach statistical significance (*p* = 0.145). Other symptoms, including fever, chills, dyspnea, cardiac manifestations, digestive symptoms, and skin lesions, showed no significant differences between groups.

Multivariable analyses of discharge outcomes were also performed. However, given the limited sample size of patients, these findings should be interpreted with caution, as effect estimates may be unstable and confidence intervals wide. The results of this analysis are reported in Table 4.

A discussion of the decision-tree performance and structure follows bellow; these findings should be interpreted with caution given the limited sample size of patients.

A cost-complexity-pruned CART model was developed to delineate patients at risk of in-hospital death. Six routinely collected baseline variables—hemodialysis status (HD), age, sex, total comorbidity count, chronic hypotension (hTA), and length of stay (LOS)—were entered a priori. Hyper-parameters were tuned by fivefold stratified cross-validation (depth 2–4, minimum leaf size 2–6, α 0–0.01), maximizing balanced accuracy; the optimal tree had a depth of 4, a minimum of four observations per terminal node, and α = 0.

The final structure contained four decision levels (12 terminal nodes) and achieved a cross-validated balanced accuracy of 0.73. Variable importance ranked hemodialysis as the dominant discriminator, followed sequentially by comorbidity load, LOS, and age; sex and chronic hypotension were pruned out.

• Root split—HD: Non-dialyzed patients followed the left branch and exhibited a low mortality rate (15%), whereas dialyzed patients proceeded to the right branch for further stratification.
• Second level—comorbidity burden (cut-point >4 diagnoses): HD patients with ≤4 comorbidities had an intermediate mortality of 21%; those with >4 underwent additional partitioning.
• Third level—LOS (≤14 d): Short admissions within the high-comorbidity HD subgroup were ominous, prompting a final age split.
• Terminal split—age (>72 y): The combination of HD, >4 comorbidities, short LOS, and age > 72 years defined the worst-prognosis phenotype, with an observed mortality of 80% (8 deaths/10 cases).

Conversely, HD patients aged ≤63 years, or those with LOS > 14 days despite multiple comorbidities, showed mortality below one third. The tree’s Hosmer–Lemeshow calibration test (*p* = 0.71) and Brier score (0.14) confirmed acceptable goodness-of-fit.

### 3.1. Microbiological Examinations Results

Among the 56 patients enrolled in our study, at least one microbiology specimen was collected for every patient, and 46/56 (82.1%) had ≥1 positive result. The cohort-level yields (n = 56) were as follows: blood 46.4%, urine 37.5%, pharyngeal swab 23.2%, tracheal aspirate 12.5%, sputum 8.9%, catheter tip 7.1%, wound secretion 7.1%, and ≤1.8% for stool, CSF, meningitis/encephalitis panel, and pleural fluid. Details regarding the harvested specimens are presented in the following table (Table 5).

Across all positive specimens, 98 isolates were identified. *S. aureus* predominated (25/98, 25.5%), followed by *Klebsiella pneumoniae* (19/98, 19.4%) and *E. coli* (15/98, 15.3%). Other Enterobacterales included *Proteus mirabilis* (n = 7), *Klebsiella aerogenes* (n = 1), *K. oxytoca* (n = 1), *Morganella morganii* (n = 1), and *Proteus* spp. (n = 2), for a combined amount of Enterobacterales isolates of 46/98 (46.9%). Non-fermenters were uncommon (*A. baumannii*, n = 4; *Pseudomonas aeruginosa*, n = 2). Gram-positive non-staphylococci were rare (*Enterococcus faecium*, n = 2; *E. faecalis*, n = 1; *Corynebacterium striatum*, n = 2; *C. urealyticum*, n = 1). Fungal and other detections were infrequent (*Candida* spp., n = 5; *Cryptococcus neoformans*, n = 2; *Aspergillus fumigatus*, n = 1), alongside single entries such as *Clostridioides difficile* and HHV-6.

From the electronic records we retrieved antibiotic susceptibility test results, with the aim of analyzing them for 61 clinical isolates. Three species accounted for 75.4% of identifications: *Staphylococcus aureus* (n = 21, 34.4%), *Klebsiella pneumoniae* (n = 15, 24.6%), and *Escherichia coli* (n = 10, 16.4%). Isolates were most frequently recovered from blood cultures (n = 21, 34.4%), followed by tracheal aspirates (n = 10, 16.4%), urine cultures (n = 9, 14.8%), and pharyngeal swabs (n = 8, 13.1%); additional sources included sputum (n = 4, 6.6%), catheter tips (n = 2, 3.3%), and single isolates from wound, fistula, and pleural fluid. Among commonly tested agents, the susceptibility rates (with the number of tests in parentheses) were as follows: gentamicin 72.2% (54), ciprofloxacin 56.5% (46), piperacillin–tazobactam 55.6% (36), cefepime 59.4% (32), ceftazidime 51.4% (37), and cefotaxime 50.0% (32).

Regarding *Staphylococcus aureus* (n = 21), oxacillin resistance (MRSA) was 38.1% (8/21; Wilson 95% CI, 20.8–59.1). Susceptibility was universal where tested for vancomycin (13/13), teicoplanin (13/13), linezolid (16/16), levofloxacin (12/12), ciprofloxacin (9/9), and trimethoprim–sulfamethoxazole (19/19). Clindamycin susceptibility was 52.4% (11/21), with inducible clindamycin resistance (D-test) detected in 38.1% (8/21; 95% CI, 20.8–59.1). Tetracycline susceptibility was 47.6% (10/21).

Regarding *Klebsiella pneumoniae* (n = 15) patterns of resistance, susceptibility to third-/fourth-generation cephalosporins was 33.3% for cefotaxime (5/15), ceftazidime (5/15), and cefepime (5/15). Piperacillin–tazobactam susceptibility was 40.0% (6/15). Fluoroquinolone activity was limited (ciprofloxacin 30.8%, 4/13; levofloxacin 25.0%, 1/4). Aminoglycosides showed intermediate performance (gentamicin 53.3%, 8/15, with 33.3% intermediate; amikacin 46.7%, 7/15). Carbapenems demonstrated modest activity in small test sets (imipenem 25.0%, 1/4; meropenem 40.0%, 2/5; ertapenem 40.0%, 2/5). Ceftazidime–avibactam susceptibility was 42.9% (6/14). Colistin susceptibility was 80.0% (4/5).

Regarding the *Escherichia coli* (n = 10) susceptibility test results, extended-spectrum cephalosporin susceptibility was 60.0% for cefotaxime (6/10) and ceftazidime (6/10), and 85.7% for cefepime (6/7). Piperacillin–tazobactam susceptibility was 70.0% (7/10). Fluoroquinolones were less active (ciprofloxacin 44.4%, 4/9; levofloxacin 57.1%, 4/7). The susceptibility of the aminoglycosides was as follows: amikacin 100% (6/6) and gentamicin 60.0% (6/10). Carbapenems remained 100% susceptible where tested (imipenem 3/3; meropenem 5/5; ertapenem 4/4). For urinary agents, nitrofurantoin and fosfomycin were 100% susceptible (5/5 and 4/4, respectively). Ceftazidime–avibactam was 100% susceptible (9/9).

**Figure 2:**
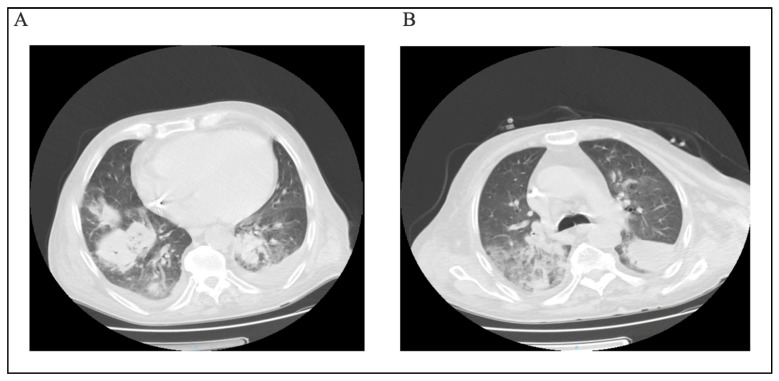
(**A**,**B**) CT scan section of patient with *Cryptococcus neoformans* infection.

We also retrieved six fungal isolates, *Candida* spp. (n = 3), *Candida tropicalis* (n = 1), *Aspergillus fumigatus* (n = 1), and *Cryptococcus neoformans* (n = 1), predominantly from tracheal aspirates (3/6), with single isolates from urine, cerebrospinal fluid (CSF), and a catheter tip. Antifungal susceptibility testing (AFST) was available for three isolates (two *Candida* spp. and one *C. tropicalis*). Among isolates with AFST, all agent–organism pairs were categorized as susceptible: for *Candida* spp., this included fluconazole 2/2, caspofungin 2/2, micafungin 2/2, and voriconazole 1/1; for *C. tropicalis*, amphotericin B 1/1, caspofungin 1/1, micafungin 1/1, fluconazole 1/1, and voriconazole 1/1. Given the very small denominators (often n = 1–2) and incomplete testing, these findings are descriptive and should be interpreted with caution. The discharge disposition rates within this fungal subset were 2/6 deceased and 4/6 cured. Notably, one patient diagnosed with pulmonary aspergillosis presented with concurrent cryptococcal meningoencephalitis and human herpesvirus 6, ultimately resulting in an unfavorable outcome. Pulmonary CT scan images of this case were included for reference purposes (see Figure 2).

### 3.2. Treatment

Most patients received antibiotic therapy (49/56, 87.5%). Systemic corticosteroids were administered to one third of patients (19/56, 33.9%; dexamethasone 17/56, 30.4%; methylprednisolone 2/56, 3.6%), antifungals to 16/56 (28.6%), and antivirals to 11/56 (19.6%; oseltamivir 3/56, 5.4%; favipiravir 5/56, 8.9%; remdesivir 3/56, 5.4%).

Antibiotic therapy was administered to 49/56 patients (87.5%). For clarity, we define monotherapy as exposure to one antibacterial class during the index admission and combination therapy as exposure to ≥2 antibacterial classes during the same admission (classes may have been sequential or overlapping; same-day co-administration was not specifically quantified). Among antibiotic-treated patients, 36/49 (73.5%) received combination therapy and 13/49 (26.5%) received monotherapy. Across the cohort, the most frequently used classes were cephalosporins (25/56, 44.6%), oxazolidinones (linezolid; 20/56, 35.7%), and fluoroquinolones (17/56, 30.4%), followed by tetracyclines (12/56, 21.4%), aminoglycosides (9/56, 16.1%), carbapenems (6/56, 10.7%), glycopeptides (6/56, 10.7%), polymyxins (5/56, 8.9%), and sulfonamides (3/56, 5.4%); daptomycin was not used. Use patterns differed by HD status (non-HD n = 27; HD n = 29): cephalosporins were more common in non-HD (17/27, 63.0%) than HD (8/29, 27.6%), whereas carbapenems (5/29, 17.2% vs. 1/27, 3.7%), glycopeptides (5/29, 17.2% vs. 1/27, 3.7%), aminoglycosides (7/29, 24.1% vs. 2/27, 7.4%), and tetracyclines (8/29, 27.6% vs. 4/27, 14.8%) were relatively more common in HD patients; fluoroquinolone use was similar (8/29, 27.6% vs. 9/27, 33.3%) (see Table 6). These differences are non-causal, unadjusted, and likely reflect confounding by indication (i.e., sicker patients receiving broader therapy).

## 4. Discussion

In this single-center retrospective cohort of 56 hospitalized patients with end-stage CKD and infection (HD, n = 30; non-HD, n = 26), univariable analyses demonstrated substantially higher in-hospital mortality among HD patients (46.7% vs. 15.4%; risk ratio 3.03, 95% CI 1.14–8.08), whereas length of stay, use of advanced ventilatory support, and most presenting features were comparable between groups. Laboratory findings reflected expected renal-replacement physiology in the HD group (higher creatinine with modest electrolyte shifts), and symptom profiles differed modestly (lower prevalence of cough and urinary manifestations in HD). In multivariable logistic regression, hemodialysis remained the dominant independent predictor of death (adjusted OR 38.22, 95% CI 1.55–940.53), alongside hypotension and male sex, with good apparent discrimination (AUC 0.867; optimism-corrected 0.803) and acceptable calibration. Microbiological yield was high and was dominated by *Staphylococcus aureus*, *Klebsiella pneumoniae*, and *Escherichia coli*.

Sepsis is particularly associated with hemodialysis, especially in the first year after admission. It is connected to the risk of myocardial infarction, heart failure exacerbation, peripheral venous disease, or stroke [15], association that is responsible for a greater risk of mortality. Bacteremia in hemodialysis patients is most commonly caused by *Staphylococcus aureus*, with an increase in MRSA, VRSA strains, and coagulase-negative staphylococci, followed by enterococci and Gram-negative bacilli [43,44,45,46,47]. Sepsis associated with central venous catheters necessitates the replacement of the existing catheter with an alternative venous access route. This requirement stems from the ineffectiveness of antibiotic therapy against biofilms formed on the catheter surface, coupled with the risks of sustained bacteremia, emergence of septic metastases, and increased mortality. Moreover, the biofilm’s presence, characterized by a high bacterial density and the employment of ‘quorum sensors’, facilitates bacterial interactions with the transcription activators LasR and RhlR. This interaction leads to the augmented expression of resistance genes and enhanced production of catalases, proteases, and superoxide dismutase, thereby shielding bacteria from both neutrophil action and antibiotic effects. Additionally, the biofilm on the venous catheter promotes colonization by *Staphylococcus aureus*, which adheres to fibronectin, and coagulase-negative staphylococci, which directly adhere to polymer surfaces. In the cases documented in this study, all systemic infections with *Staphylococcus aureus* were associated with the presence of a central venous catheter (tunneled CVCs) within the first year, as well as the case involving *Corynebacterium striatum*, where the CVC had been placed for one month and was associated with an endocardial lesion on the mitral valve.

The indiscriminate use of antibiotics occasionally leads to the development of bacterial multidrug resistance, encompassing organisms such as MRSA, VRE, and MDR Gram-negative bacilli [48]. This proliferation of resistance compromises the effectiveness of standard first-line treatments, which, although they are characterized by an ostensibly ultra-broad spectrum, are inappropriately aligned with the local microbial ecology [48].

In a study conducted by Rteil et al. [49], coagulase-negative *Staphylococcus* was identified as the leading pathogen in 49% of bacteremia cases among hemodialysis patients, followed by *Staphylococcus aureus* and *Escherichia coli*. Mortality was predominantly associated with infections caused by multidrug-resistant (MDR) bacteria, the requirement for mechanical ventilation, or admission to an Intensive Care Unit. Similar data were obtained by Berman et al., who conducted a retrospective study on hemodialysis patients [50]. The primary cause of infection in this population was coagulase-negative *Staphylococcus*, followed by *Staphylococcus aureus*. Regarding the resistance profiles, MRSA is notably prevalent, followed by VRE and MDR Gram-negative bacilli [48].

The second leading cause of infection-related morbidity in hemodialysis patients is pneumonia, encompassing both bacterial and viral forms (including influenza and, during the COVID-19 pandemic, severe pneumonia associated with the Delta variant in these patients) and mixed viral–bacterial infections. These patients are at 16–17 times higher risk of death than that of the general population [20]. The increased risk of pneumonia can be explained by potential central sleep apnea, hypotonia of the striated muscles, hyperhydration syndrome, possible uremic pneumonitis or pleuritis, pulmonary calcifications, and hypoxemia during hemodialysis sessions. In our cohort of patients with end-stage chronic kidney disease that also required hemodialysis, pneumonia cases were outnumbered by urinary tract infections (eight cases compared to five cases of pleuropulmonary infections); however, the pneumonia cases were notable for their severity, often requiring mechanical ventilation or admission to intensive care units, and were caused by XDR (extensively drug-resistant) or PDR (pan-drug-resistant) bacteria. Other studies have confirmed an increased risk of infectious endocarditis in hemodialysis patients, rising from 7% to 20%, particularly caused by Staphylococcus aureus, which has shown an increase from 10% to 68% [15]. In the United States, there are 483 episodes per year per 100,000 patients compared to the general population, where seven episodes per 100,000 are diagnosed annually [51]. In our study, we encountered a single case of infectious endocarditis caused by *Corynebacterium striatum*.

Our study had a few limitations that warrant discussion. Since this was a retrospective analysis, it might not provide a full overview of the situation. Furthermore, as this is a single-center study, it may include also some heterogeneity regarding the included data. Additionally, the number of enrolled patients is relatively small. This cohort does not reflect a nationally diverse sample of hospitals; rather, it comprises patients from our county and adjacent counties. Finally, we analyzed patients from a restricted time window; therefore, there is a possibility of residual confounding in our methodology. Despite these limitations, the study has several strengths. We leveraged a comprehensive database capturing a wide range of clinical and laboratory variables collected at admission and throughout hospitalization.

## 5. Conclusions

In this single-center cohort of 56 hospitalized patients with end-stage CKD and infection, maintenance hemodialysis was independently associated with higher in-hospital mortality. Hypotension and male sex emerged as additional independent risk factors. Length of stay, use of advanced ventilatory support, and most presenting features did not differ meaningfully by dialysis status, and laboratory differences largely reflected renal replacement physiology rather than systemic severity. These findings identify dialyzed patients as a high-risk subgroup that may benefit from early risk stratification, vigilant hemodynamic management, and aggressive, stewardship-guided infection control. Infections associated with hemodialysis are predominantly characterized by bacteremia, which is most often caused by MRSA and is especially frequent in the first year of HD via tunneled central venous catheters (CVCs), urinary infections with MDR Gram-negative bacilli, and pneumonia also associated with XDR or PDR Gram-negative bacteria. The mortality rate was 50%, attributable to the immunosuppression in these patients as well as the limited therapeutic options due to the susceptibility of the isolated pathogens. It is essential to be aware of local resistance patterns to guide appropriate therapeutic strategies and to employ rapid diagnostic techniques for etiological determination and rapid identification of bacterial multidrug resistance. Given the retrospective, single-center design and limited events-per-variable, confirmation in larger, prospective multicenter studies is warranted.

## Figures and Tables

**Table 1:** Characteristics of the study population.

Characteristic	HD Group (N = 30)	Non-HD Group (N = 26)	*p* (Pearson Chi-Square)
Age in years, mean ± SD	65.47 ± 17.07	74.31 ± 13.17	0.472
Male gender (n, %)	18, 60%	12, 46.15%	0.300
Urban area residence (n, %)	16, 53.33%	14, 53.84%	≥0.10
Length of stay in days, mean ± SD, 95% CI, Median	14.7 ± 13.1, 9.4–20.0, 10	15.0 ± 15.1, 9.3–20.6; 10.5	≥0.55
Hypertension (n, %)	23, 76.66%	10, 38.46%	≥0.10
Other cardiac pathology (n, %)	25, 83.33%	11, 42.30%	≥0.10
Solid tumors (n, %)	3, 10%	2, 7.69%	≥0.10
Hematologic disorders (n, %)	3, 10%	1, 3.84%	≥0.10
Urological pathology (n, %)	3, 10%	1, 3.84%	0.056
Psychiatric pathology (n, %)	3, 10%	0, 0.0%	0.056
Prior neurological event (n, %)	9, 30%	0, 0.0%	≥0.10
Type 2 diabetes mellitus (n, %)	11, 36.66%	0, 0.0%	≥0.10
Other endocrine disorders (n, %)	6, 20%	0, 0.0%	≥0.10
Nutritional status			
BMI 18.5–24.9 kg/m^2^ (n, %)	14, 46.66%	16, 61.53%	0.240
BMI 25–29.9 kg/m^2^ (n, %)	4, 13.33%	5, 19.23%	
BMI > 30 kg/m^2^ (n, %)	12, 40%	5, 19.23%	
Access site for HD			
AV fistula (n, %)	20, 66.66%	-	-
Tunneled CVCs (n, %)	10, 33.33%	-	
Status at the time of discharge			
Favorable outcome (n, %)	16, 53.33%	22, 84.61%	0.012
Unfavorable outcome (deceased) (n, %)	14, 46.66%	4, 15.38%	
O_2_ therapy during hospitalization			
Oxygen mask (n, %)	8, 26.66%	6, 23.07%	0.353
High-flow nasal oxygen (n, %)	2, 6.66%	0, 0.0%	0.180
Continuous positive airway pressure (n, %)	2, 6.66%	0, 0.0%	0.180
Orotracheal intubation and mechanical ventilation (n, %)	9, 30%	4, 15.38%	0.196

HD—hemodialysis; SD—standard deviation; BMI—body mass index; AV—arteriovenous; CVC—central venous catheter.

**Table 2:** Laboratory test performed during hospitalization.

Hemodialysis	No		Yes		*p* (Mann–Whitney U Test, 2-Tailed)	Total (n = 56)	
	Mean	Std. Deviation	Mean	Std. Deviation		Mean	Std. Deviation
Urea min. level	79.50	31.82	120.00	66.46	0.457	78.60	41.32
Creatinine min. level	2.59	0.23	3.40	1.37	**0.001**	2.91	1.93
Urea max. level	200.50	149.20	226.00	82.02	0.197	132.63	62.55
Creatinine max. level	4.67	2.81	7.14	2.46	**0.001**	7.24	16.31
Aspartate aminotransferase	1987.50	2772.56	20.00	9.89	**0.004**	129.64	537.04
Alanine aminotransferase	496.70	660.43	40.50	36.06	0.191	63.26	147.48
Sodium	128.00	9.89	138.50	4.95	**0.044**	138.65	5.84
Potassium	4.99	0.69	5.14	1.04	**0.008**	4.4193	0.92
LDH	601.00	458.20	337.77	69.26	0.129	327.86	195.84
CPK	48.00	15.55	40.00	12.72	0.823	208.68	339.31
Amylase	48.50	38.89	38.50	14.84	0.678	85.94	107.22
C-reactive protein	117.85	98.90	226.30	173.88	0.412	206.06	158.08
Fibrinogen	555.07	157.65	577.48	348.72	0.697	592.21	299.27
Erythrocyte sedimentation rate	47.56	28.18	40.23	39.52	0.275	42.13	35.72
Procalcitonin	8.54	23.94	9.90	21.61	0.385	22.57	98.20
Neutrophil to Lymphocyte Ratio	36.61	32.50	23.54	2.41	0.857	18.35	15.00

LDH—Lactate Dehydrogenase; CPK—Creatine Phosphokinase.

**Table 3:** Clinical manifestations at the time of admission.

Crosstabulation				
		Hemodialysis		
		No	Yes	Total
**Fever**	**No**	7	13	20
	**Yes**	19	17	36
**Chi-Square Tests**	Asymptotic Significance (2-sided)			
Pearson	0.201			
**Chills**	**No**	12	18	30
	**Yes**	14	12	26
**Chi-Square Tests**	Exact Sig. (2-sided)			
Fisher’s Exact Test	0.421			
**Cough**	**No**	9	19	28
	**Yes**	17	11	28
**Chi-Square Tests**	Asymptotic Significance (2-sided)			
Pearson	0.032			
**Dyspnea**	**No**	11	20	31
	**Yes**	15	10	25
**Chi-Square Tests**	Exact Sig. (2-sided)			
Fisher’s Exact Test	0.106			
**Cardiac Manifestations**	**No**	15	23	38
	**Yes**	11	7	18
**Chi-Square Tests**	Exact Sig. (2-sided)			
Fisher’s Exact Test	0.159			
**Urinary manifestation**	**No**	17	27	44
	**Yes**	9	3	12
**Chi-Square Tests**	Exact Sig. (2-sided)			
Fisher’s Exact Test	0.047			
**Digestive Manifestations**	**No**	18	21	39
	**Yes**	8	9	17
**Chi-Square Tests**	Exact Sig. (2-sided)			
Fisher’s Exact Test	1.000			
**Skin Lesions**	**No**	21	26	47
	**Yes**	5	4	9
**Chi-Square Tests**	Exact Sig. (2-sided)			
Fisher’s Exact Test	0.719			
**Neurological Manifestations**	**No**	18	15	33
	**Yes**	8	15	23
**Chi-Square Tests**	Asymptotic Significance (2-sided)			
Pearson	0.145			
**Crosstabulation**				
		**Hemodialysis**		
		**No**	**Yes**	**Total**
**Fever**	**No**	7	13	20
	**Yes**	19	17	36
**Chi-Square Tests**	Asymptotic Significance (2-sided)			
Pearson	0.201			
**Chills**	**No**	12	18	30
	**Yes**	14	12	26
**Chi-Square Tests**	Exact Sig. (2-sided)			
Fisher’s Exact Test	0.421			
**Cough**	**No**	9	19	28
	**Yes**	17	11	28
**Chi-Square Tests**	Asymptotic Significance (2-sided)			
Pearson	0.032			
**Dyspnea**	**No**	11	20	31
	**Yes**	15	10	25
**Chi-Square Tests**	Exact Sig. (2-sided)			
Fisher’s Exact Test	0.106			
**Cardiac Manifestations**	**No**	15	23	38
	**Yes**	11	7	18
**Chi-Square Tests**	Exact Sig. (2-sided)			
Fisher’s Exact Test	0.159			
**Urinary manifestation**	**No**	17	27	44
	**Yes**	9	3	12
**Chi-Square Tests**	Exact Sig. (2-sided)			
Fisher’s Exact Test	0.047			
**Digestive Manifestations**	**No**	18	21	39
	**Yes**	8	9	17
**Chi-Square Tests**	Exact Sig. (2-sided)			
Fisher’s Exact Test	1.000			
**Skin Lesions**	**No**	21	26	47
	**Yes**	5	4	9
**Chi-Square Tests**	Exact Sig. (2-sided)			
Fisher’s Exact Test	0.719			
**Neurological Manifestations**	**No**	18	15	33
	**Yes**	8	15	23
**Chi-Square Tests**	Asymptotic Significance (2-sided)			
Pearson	0.145			

**Table 4:** Multivariable analyses report.

Predictor	Coding/Unit	Adjusted OR	95% CI	*p*-Value
Primary multivariable logistic regression (outcome: death at discharge)				
Hemodialysis (HD)	Yes vs. No	38.22	1.55–940.53	0.026
Hypotension	Yes vs. No	17.55	1.46–210.92	0.024
Sex	Male vs. Female	4.41	1.29–15.11	0.018
Age	Per year	1.03	0.99–1.08	0.171
Comorbidity count	Per diagnosis	1.32	0.91–1.92	0.150
Length of stay (LOS)	Per day	0.94	0.84–1.04	0.206
Sensitivity model (augmented with log2 maximum creatinine; interpretation per doubling)				
Hemodialysis (HD)	Yes vs. No	49.17	1.56–1550.24	0.027
Hypotension	Yes vs. No	20.03	1.31–305.13	0.031
Sex	Male vs. Female	4.34	1.19–15.90	0.027
Max creatinine (log2)	Per doubling	0.83	0.50–1.38	0.480

Model performance/validation (primary model): N = 56; events = 18 (32.1%). Global model test *p* = 0.0006. AUC 0.867. Bootstrap internal validation (1000 resamples): mean optimism 0.063 → optimism-corrected AUC 0.803 (bootstrap 95% CI for test AUC 0.760–0.876). Model performance (sensitivity): AUC 0.865.

**Table 5:** Types of harvested specimens.

Type of Harvested Specimens	No. of Harvested Specimens	No. of Positive Samples	% of Positive Samples	95% CI
Blood	32	26	81.2%	64.7–91.1
Tracheal aspirate	9	7	77.8%	45.3–93.7
Urine	32	21	65.6%	48.3–79.6
Sputum samples	10	5	50.0%	23.7–76.3
Pharyngeal swab	38	13	34.2%	21.2–50.1
Catheter tip	4	4	100%	-
Wound secretions	6	4	66.7%	-
Stool samples	8	1	12.5%	-

**Table 6:** Key antibacterial classes used overall and by hemodialysis status (with Fisher’s exact *p*).

Class	Overall n/N (%)	Non-HD n/27 (%)	HD n/29 (%)	*p* (HD vs. Non-HD)
Carbapenems	6/56 (10.7)	1/27 (3.7)	5/29 (17.2)	0.195
Fluoroquinolones	17/56 (30.4)	9/27 (33.3)	8/29 (27.6)	0.773
Tetracyclines	12/56 (21.4)	4/27 (14.8)	8/29 (27.6)	0.334
Aminoglycosides	9/56 (16.1)	2/27 (7.4)	7/29 (24.1)	0.146
Glycopeptides	6/56 (10.7)	1/27 (3.7)	5/29 (17.2)	0.195

## Data Availability

The original contributions presented in this study are included in the article. Further inquiries can be directed to the corresponding authors.

## Abbreviations

The following abbreviations are used in this manuscript:

CVC	Central venous catheters
HD	Hemodialysis
CKD	Chronic kidney disease
BMI	Body mass index
CRP	C-reactive protein
CART	Classification and regression tree
